# Effect of transcription terminator usage on the establishment of transgene transcriptional gene silencing

**DOI:** 10.1186/s13104-018-3649-2

**Published:** 2018-07-28

**Authors:** Ana Pérez-González, Elena Caro

**Affiliations:** Centre for Plant Biotechnology and Genomics, Universidad Politécnica de Madrid (UPM)-Instituto Nacional de Investigación y Tecnología Agraria y Alimentaria (INIA), Campus Montegancedo UPM, Pozuelo de Alarcón, 28223 Madrid, Spain

**Keywords:** Arabidopsis, Transgene, Terminator, DNA methylation, TGS, PTGS, RdDM, Silencing

## Abstract

**Objective:**

Obtaining high and stable transgene expression is of vital importance for plant genetic engineering. A lot is known about the relationship between terminator efficiency and gene expression, but no studies have addressed the relationship between terminator usage and transgene expression stability or heritable gene silencing. In this paper, we aim to analyze if terminators are a determining factor in the establishment of promoter DNA methylation of plant transgenes.

**Results:**

Our experiments comparing plants with a LUC reporter under the 35S CaMV promoter and good efficiency terminators (Thsp, T35S) show that the use of efficient terminator sequences does not avoid the accumulation of promoter DNA methylation and transgene silencing. However, Thsp lead to a higher reporter gene expression and lower promoter DNA methylation levels than T35S, supporting that terminator usage is indeed involved in the establishment of TGS by methylation of transgenes’ promoters. In the case of a terminatorless construct, the PTGS initiated by the improperly terminated mRNAs is not followed by the establishment of heritable silencing in the form of strong promoter DNA methylation, like in the case of TAS genes and reactivated TEs (for the transgene DNA methylation levels remained below the 20%).

**Electronic supplementary material:**

The online version of this article (10.1186/s13104-018-3649-2) contains supplementary material, which is available to authorized users.

## Introduction

Since the rise of plant biotechnology, scientists have faced the important challenge to increase and stabilize transgene expression. It is known that mRNA 3′ end formation is essential for the expression of genes and that the use of different terminators can increase transgene expression in different plant species like tobacco [1], Arabidopsis [2], rice [3] and soybean [4].

Transgenes designed for overexpression often induce RNA silencing and it has been proposed that it might be triggered by the production of improperly terminated and unpolyadenylated mRNA transcripts that become templates for RNA dependent RNA polymerase 6 (RDR6) generating dsRNA which will be processed into siRNAs [5] and guide homologous mRNA cleavage or translational repression, a mechanism known as post-transcriptional gene silencing (PTGS) [6]. Consistent with this, proper placement of a termination signal after a terminatorless transgene and the use of a double terminator can account for the enhancement and stabilization of transgene expression, both because of the abolishment of PTGS [6, 7].

However, little is known about how transgenes initiate transcriptional gene silencing (TGS). TGS involves decreased RNA synthesis because of promoter methylation and/or chromatin condensation as a consequence of small RNAs targeting DNA sequences for cytosine methylation, a phenomena termed RNA directed DNA methylation (RdDM) [8, 9]. In the last few years, it has been hypothesized that the siRNAs produced through PTGS can participate in RdDM initiation [10]. This pathway was first identified at TAS genes [11] and later found to target transcriptionally active transposons [12]; however, its existence in transgenes remains unclear.

To analyze if terminator usage is a determining factor in the establishment of TGS of transgenes, we generated lines with a LUC reporter followed by either good efficiency terminators (Thsp, T35S) or no terminator at all and compared the expression and methylation acquired in the transgene.

## Main text

### Methods

#### Generation of transformation plasmids

GoldenBraid2.0 (GB) cloning system was used for cloning. Plasmids GB0030, GB0036, GB0035, GB0235 and GB0466 were used for assembly. Firefly luciferase was amplified from GB0255 using primers 1678 (5′-GCGCCGTCTCGCTCGAATGGAAGACGCCAAAAACATAAAG-3′)/1679 (5′-GCGCCGTCTCGCTCGCTGCTTACACGGCGATCTTTCCGC-3′). FUS3short was amplified from Arabidopsis genomic DNA using primers 1668 (5′-GCGCCGTCTCGCTCGGCAGGGAAATGTTCTTACTATTATCCAGTCAT-3′)/1676 (5′-GCGCCGTCTCGCTCGAAGCTTATCCACCCAAAAAATCGAG-3′) to include FUS3 AAs 246–283. Phusion Hot Start II DNA Polymerase (ThermoFisher) was used for DNA amplification.

Restriction-ligation reactions were set up as described previously [13]. BsmBI (ThermoFisher), BsaI and BtgZI (New England BioLabs) and T4 Ligase (Promega) were used. ffLUC and FUS3short were domesticated into pUPD vectors using BsmBI enzyme. p35S, ffLUC, FUS3short and Thsp or T35S in pUPD vectors were assembled into pDBG_2alpha2. These three constructions were assembled with GB0235 into pDGB_2omega1, and again assembled with GB0466, a “twister” plasmid that contains a 150 bp stuffer fragment in order to get the constructions into pDBG_2alpha1 as the final plasmid.

After digestion/ligation, 10 µl of reaction were transformed into *E. coli* DH5α chemically competent cells. Positive clones were selected in LB solid media containing Ampicillin (100 µg/ml) (Formedium), Spectinomycin (50 µg/ml) (Sigma) or Kanamycin (50 µg/ml) (Formedium), X-Gal (20 µg/ml) (Duchefa) and IPTG (1 mM) (Anatrace). Plasmid DNA was extracted using GenElute™ Plasmid Miniprep Kit (Sigma-Aldrich).

#### Plant transformation

Transformation plasmids were introduced into *Agrobacterium tumefaciens* C58 quimiocompetent cells and plated in LB medium supplemented with Rifampicin (25 µg/ml) (Sigma-Aldrich) and Kanamycin (50 µg/ml). A single transformant colony was grown in 200 mL LB medium supplemented with the same antibiotics at 28 °C under constant shaking to perform *Arabidopsis thaliana* Col0 plant transformation [13]. T1 seeds were plated in MS medium [14] with 1% glucose and supplemented with Hygromycin B (15 µg/ml) (Formedium) for selection of transformants.

#### Plant growth conditions

*Arabidopsis thaliana* Columbia-0 accession (Col-0) seedlings were grown in plates in MS medium with 1% sucrose in Corning^®^ square bioassay dishes (Sigma-Aldrich) in a growth chamber under 16/8 h light/dark conditions at 22 °C. 10 day-old seedlings were transferred to soil and grown in an environment controlled room (FitoClima HP, Aralab) under 16/8 h light/dark conditions, at 22 °C and 65% RH.

#### Luciferase reporter assay

For luciferase imaging, 30 seedlings per line were sowed in plates while 5 leaves from different plants were put on a plate to analyze LUC activity. d-Luciferin Firefly, potassium salt (Biosynth) was dissolved in sterile H_2_O with 0.01% Triton X-100 to a final concentration of 0.2 µM and sprayed over. After 6 min in the dark, luciferase activity was measured in a NightOWL II LB 983 (Berthold Technologies), with 3 min of exposition.

#### Bisulfite conversion and sequencing

DNA from 10 day-old seedlings and leaves of 28 day-old plants was extracted using a DNeasy Plant Mini Kit (Qiagen). Bisulfite treatment was done using the EZ DNA Methylation Gold kit (Zymo Research). Amplification from converted DNA was performed with KAPA2G Polymerase (Sigma-Aldrich) using primers 559 (5′-GAGATATATGAGAATTAAGGGAGTTAYG-3′)/560 (5′-TCAATCAAAAACTTCTCAACAAACATCRC-3′) for pNOS and 568 (5′-ATGGAGTTAAAAATTTAGATYGAGGAT-3′)/569 (5′-TCCTCTCCAAATAAAATAAACTTCCTTATATA-3′) for p35S. PCR fragments were checked on an agarose gel before the cloning step, being 288 bp for pNOS and 467 bp for p35S. 3 µl of PCR product were directly cloned into pGEM-T Easy (Promega).

White colonies were selected and plasmid DNA was extracted using GenElute™ Plasmid Miniprep Kit (Sigma-Aldrich) and 12–37 clones were sent for sequencing (Macrogen) for each promoter analysis. The efficiency of bisulfite conversion was confirmed by the observation of regions with an absence of DNA methylation. If sibling clones were sequenced, only one was included for the analysis. CyMate software [15] was used to count the converted/unconverted cytosines at each site and to calculate the percentage of methylation.

### Results and discussion

**Fig. 1:**
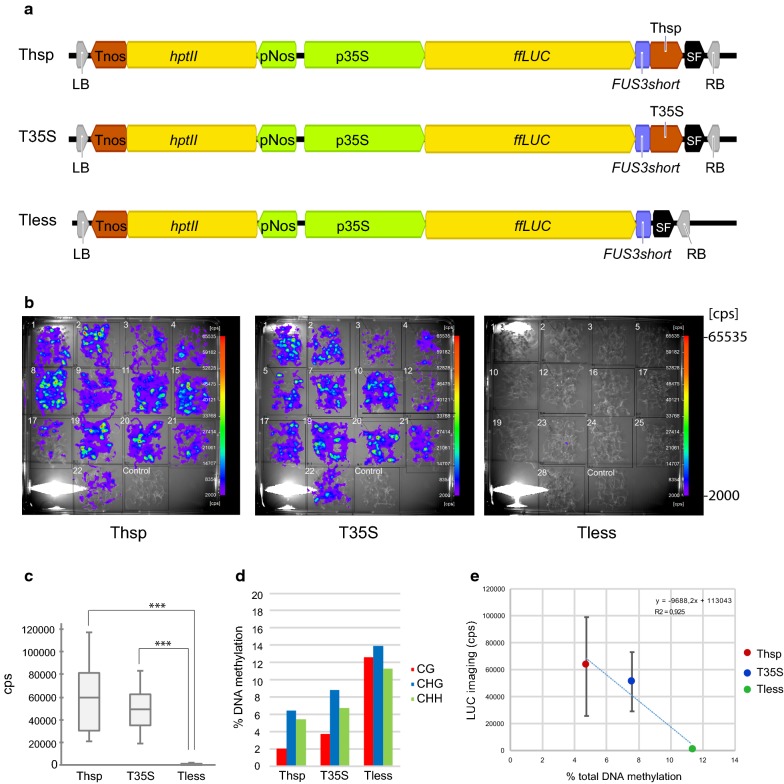
Luciferase activity and DNA methylation analysis of the 35S CaMV promoter in 10 day-old seedlings (d.o.s). **a** Schematic representation of the constructs used to transform Arabidopsis. **b** Luciferase imaging in 10 d.o.s. of T2 generation measured as counts per seconds (cps). **c** Box plot showing luciferase activity measures, represented as cps per line. *** represents highly significant differences (p < 0.001) according to Student’s test. **d** DNA methylation quantification of CG (red bars), CHG (blue bars) and CHH (green bars) cytosine contexts. **e** Luciferase activity ± standard deviation versus total DNA methylation plot for 10 d.o.s. % of DNA methylation was calculated as (number of methylated C residues in each context (CG, CHG or CHH)/total number of C residues in that context) * 100. % of total DNA methylation was calculated as (number of methylated C residues/total number of C residues) * 100. *LB* left border, *RB* right border, *Tnos* nopaline synthase terminator, *hptII* hygromycin resistance, *pNos* nopaline synthase promoter, *p35S* 35S CaMV promoter, *ffLUC* firefly luciferase, *FUS3short* FUS3 PEST sequence that includes aa 246–283, *Thsp* heat shock protein 18.2 terminator, *T35S* 35S CaMV terminator, *SF* stuffer fragment

Our goal in this work was to analyze if terminator usage is a determining factor in the establishment of DNA promoter methylation and responsible for heritable transcriptional gene silencing of transgenes. With that purpose in mind, we generated three different transgenic constructs where the CaMV 35S promoter (p35S) drove the expression of a LUC reporter gene followed either by the heat shock protein 18.2 terminator (Thsp), the CaMV 35S terminator (T35S) or no terminator at all (terminatorless construct, Tless) (Fig. 1a).

To generate a luciferase reporter with an increased rate of degradation and that responds faster to transcriptional events (like transcriptional gene silencing, the phenomenon of interest in this study) we fused luciferase to a PEST protein degradation sequence of the FUSCA3 transcription factor, previously shown to reduce 35S::GFP fluorescence by reducing GFP half-life [16].

The Thsp and the T35S are both terminators that support high levels of expression both in transient expression experiments in tobacco [1], Arabidopsis and rice protoplasts [2], and stable expression in Arabidopsis [2, 6], and tobacco transgenic plants [1]. The third construct that includes no terminator is similar to others described before to generate improperly terminated and unpolyadenylated transcripts because of read through of transcription and to trigger PTGS [6].

Thirteen *A. thaliana* T2 lines were selected and LUC activity was imaged to assess the level of transgene expression. Between the lines transformed with a same construct we could find some slight differences in LUC expression levels (Fig. 1b, Additional file 1: Figure S1), as expected from lines with transgenes inserted randomly in different positions within the genome and with different insertion copy number. However, the overall expression observed in seedlings showed that the Thsp and the T35S constructs were the ones producing the highest LUC activity and that the use of the Tless construct led to a situation not able to support translation and generation of active LUC protein (Fig. 1c).

**Fig. 2:**
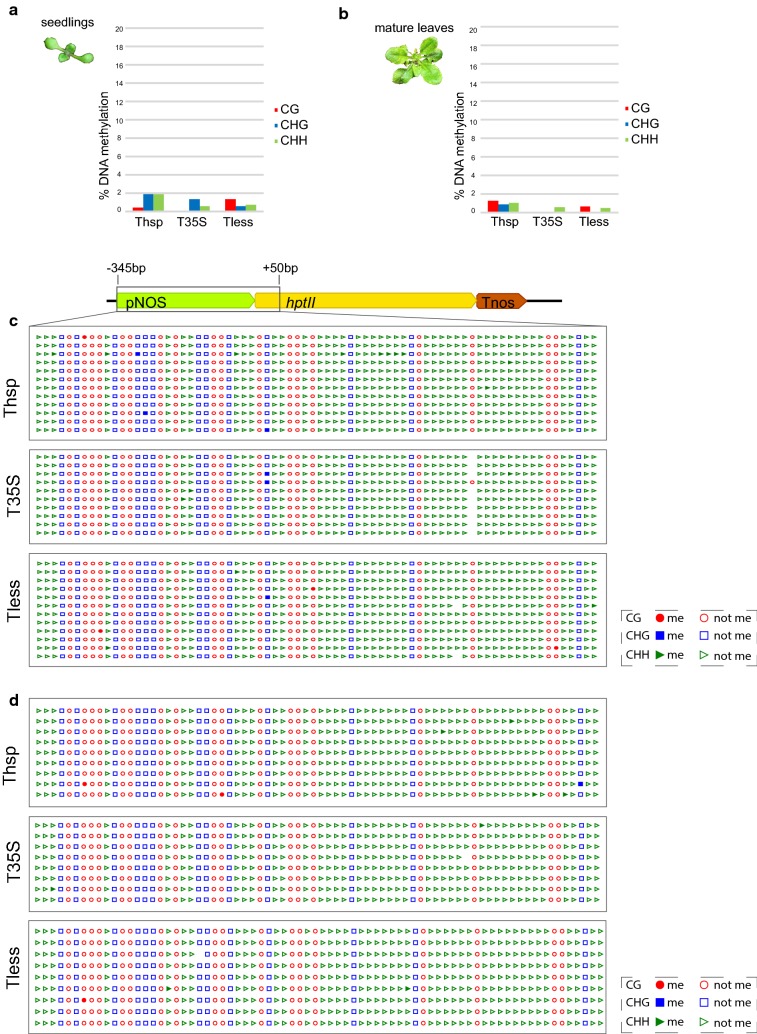
DNA methylation of the Nopaline synthase promoter. **a** DNA methylation quantification in 10 d.o.s. **b** DNA methylation quantification in mature leaves of 28 d.o.s. **c** Graphical output of the promoter methylation analysis (CyMate software) for 10 d.o.s. **d** Graphical output of the promoter methylation analysis (CyMate software) for mature leaves of 28 d.o.s. Red circles represent CG sites, blue squares represent CHG sites and green triangles represent CHH sites. Filled symbols indicate methylated cytosines while empty ones represent non methylated cytosines

To study the de novo DNA methylation status in the transgene promoter, bisulphite sequencing was carried out in 10 day-old seedlings (d.o.s.) from a pool of the 13 selected T2 lines of the three constructs. We first analyzed the modifications in the pNOS that drives the expression of the selection marker *hptII*. We could see that all lines showed a negligible level of DNA methylation, a result that indicated that neither the insertion site of the T-DNAs nor their copy number was responsible for the establishment of unspecific epigenetic silencing marks within the T-DNAs (Fig. 2a).

Next, 35S promoter modifications were measured. The levels of DNA methylation found (Fig. 1d, Additional file 2: Figure S2) were not too high but with the sequence hallmark of RdDM establishment of silencing: methylation targeted to all cytosine contexts (CG, CHG and CHH) with no clear over-representation of symmetrical methylation [17].

We could observe a strong inverse correlation between expression of the LUC transgene and promoter methylation (Perason’s correlation coefficient of − 0.962) that fits well to a linear model (Fig. 1e, R^2^ = 0.925). In the presence of efficient terminators (Thsp or T35S), the levels of DNA methylation in the promoter are not enough to avoid gene expression while, in the case of the absence of a terminator, there is a higher DNA methylation directed towards the transgene promoter and a dramatic effect over transgene expression.

It has been reported that in some cases, the p35S can become progressively methylated through vegetative growth once TGS has been initiated [18, 19] and in some other cases, silencing has been correlated with an increase in genetic dosage [20] and its effects can be observed in homozygous plants compared to hemizygous plants.

**Fig. 3:**
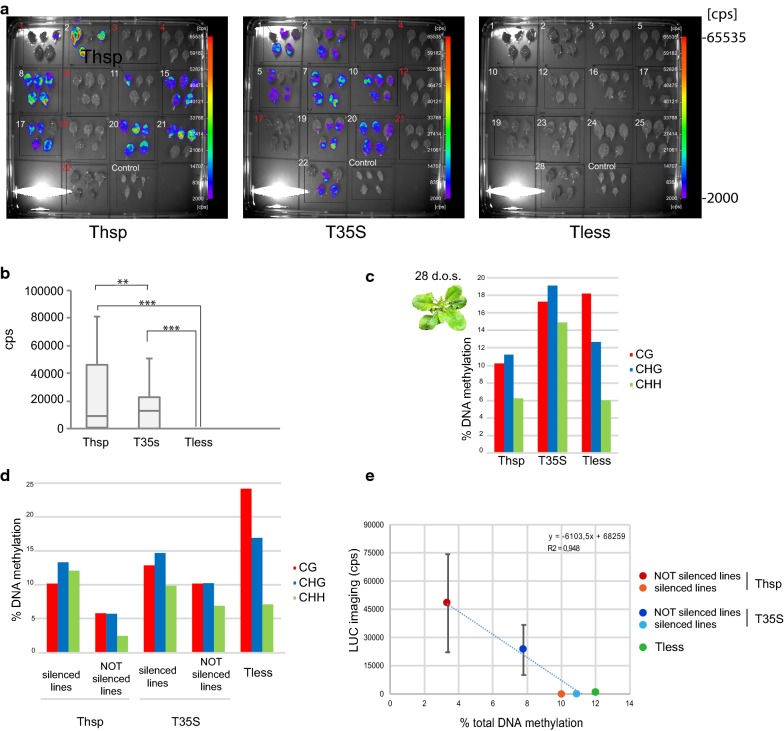
Luciferase activity and DNA methylation analysis of the 35S CaMV promoter in 28 day-old plant mature leaves. **a** Luciferase imaging of 28 day-old plant rosette leaves from the T2 generation measured as counts per seconds (cps). **b** Box plot showing luciferase activity measures, represented as cps per line. ** represents marginally significant differences (p < 0.05) and *** represents highly significant differences (p < 0.001) according to Student’s test. **c** DNA methylation quantification of a pool of mature leaves from each construction. **d** DNA methylation quantification of pools of mature leaves from silenced (number in red) and not silenced lines (number in white) from each construction. **e** Luciferase activity ± standard deviation versus total DNA methylation plot for mature leaves. % of DNA methylation was calculated as (number of methylated C residues in each context (CG, CHG or CHH)/total number of C residues in that context) * 100. % of total DNA methylation was calculated as (number of methylated C residues/total number of C residues) * 100

In our case, 28 day-old plants LUC average levels still showed that the constructs with efficient terminators were providing higher reporter expression than the Tless construct, still unable to drive LUC protein accumulation (Fig. 3a, b). However, for some efficient terminator lines, expression was very affected through plant development and showed strong silencing (Fig. 3a). Since no correlation between insert copy number (as interpreted from antibiotic resistance segregation ratios) and expression could be established (Additional file 3: Figure S3), we suggest that the appearance of silencing was a stochastic phenomenon that occurred during vegetative growth of T2 plants. Consistent with these changes in LUC expression, the transition from seedlings to mature plants was accompanied by an increase in DNA methylation levels specific for the 35S promoter (and not for the pNOS, Fig. 2b), and a change in the preferentially methylated residues, where cytosines in a symmetric context became the most abundantly methylated (Fig. 3c). This could be an indication of the transition from RdDM establishment of DNA methylation to maintenance, although the overall methylation levels achieved remained low, below the 20%.

Next, we analyzed LUC expression and DNA methylation independently in pools of leaves coming either from silenced or from not silenced lines of each construct. We could confirm that the decrease in LUC expression in the “silenced” lines was accompanied by a strong increase in all flavors of DNA methylation following a linear model (Fig. 3e, R^2^ = 0.948, Pearson’s correlation coefficient of − 0.973). In fact, whenever the total DNA methylation levels of that promoter were over 10%, LUC expression was completely abolished (Fig. 3e).

With these experiments, we have demonstrated that using good efficiency terminators like Thsp and T35S gives an advantage in expression but does not avoid the establishment of silencing and the appearance of DNA methylation in the transgene promoter (in plants transformed with both constructs, around a 40% of the T2 lines showed strong silencing). However, even after the establishment of that silencing, Thsp was able to support a stronger expression of the reporter gene and its promoter accumulated less DNA methylation.

In this report, we show that the promoter of a terminatorless construct accumulates DNA methylation levels in the same range of those of constructs with good efficiency terminators, the methylation shows preference for the CG context and as a result, LUC expression is completely abolished. The use of the terminatorless construct, while promoting the establishment of PTGS, does not lead to heritable silencing in the form of a strong promoter DNA methylation, like in the case of TAS genes [11] and reactivated transposable elements [12].

In summary, our results support the conclusion that termination efficiency does determine expression and aberrant mRNA production that can trigger PTGS, and that terminator usage can have an effect in the establishment of TGS and RdDM within transgenes, being the Thsp the terminator that supports a higher transgene expression and leads to the accumulation of lower DNA methylation levels in its promoter.

## Limitations

Only three sets of transgenic lines were compared for this analysis, two carrying good efficiency transcriptional terminators and a terminatorless construct. More information coming from other terminators would be useful, however, the differences observed support the role of terminators in TGS.

## Additional files

**Additional file 1: Figure S1.** Luciferase activity measure of the Tless lines with different scales. a) Imaging of 10 day-old seedlings. b) Imaging of leaves of 28 day-old plants.

**Additional file 2: Figure S2.** DNA methylation of the 35S CaMV promoter. a) Graphical output of the promoter methylation analysis (CyMate software) in 10 day-old seedlings. b) Graphical output of the promoter methylation analysis (CyMate software) in a pool of mature leaves from each construction. c) Graphical output of the promoter methylation analysis (CyMate software) in pools of mature leaves from silenced and not silenced lines from each construction. Red circles represent CG sites, blue squares represent CHG sites and green triangles represent CHH sites. Filled symbols indicate methylated cytosines while empty ones represent non methylated cytosines.

**Additional file 3: Figure S3.** T2 HygroB resistance segregation ratios and relationship to LUC expression. A) Data for Thsp lines. B) Data for T35S lines. C) Data for Tless lines. Note that lines with a segregation ratio < 4 (red) were considered to have only one transgenic locus.

## Abbreviations

RDR6: RNA dependent RNA polymerase 6
PTGS: post transcriptional gene silencing
TGS: transcriptional gene silencing
RdDM: RNA directed DNA methylation
Thsp: heat shock protein 18.2 terminator
T35S: CaMV 35S terminator
d.o.s.: day-old seedlings
