# Supplementary figures and images for: Effect of transcription terminator usage on the establishment of transgene transcriptional gene silencing
Source: BMC Res Notes. 2018 Jul 28;11:511. doi: 10.1186/s13104-018-3649-2 (PMC6064074; doi:10.1186/s13104-018-3649-2)

a

10 d.o.s.

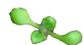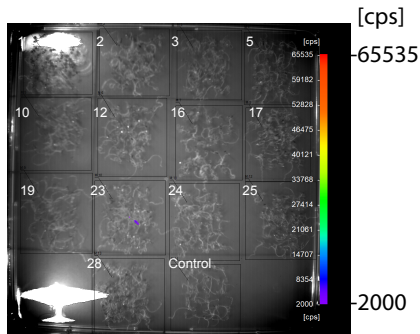

Tless

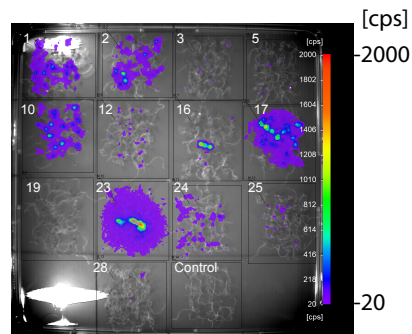

cps scale set at lower values

b

28 d.o.s.

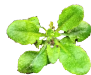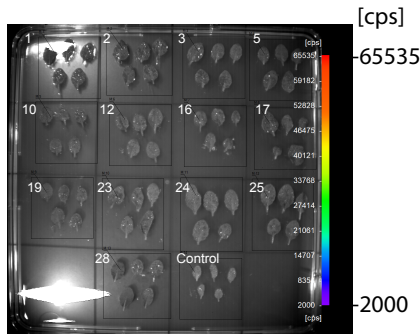

Tless

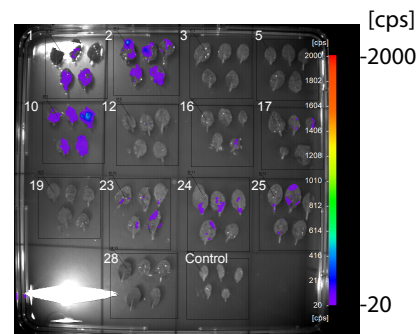

cps scale set at lower values

Supplement: Supplementary file 1 — Additional file 1: Figure S1. Luciferase activity measure of the Tless lines with different scales. a) Imaging of 10 day-old seedlings. b) Imaging of leaves of 28 day-old plants. [file 13104_2018_3649_MOESM1_ESM.pdf]

a

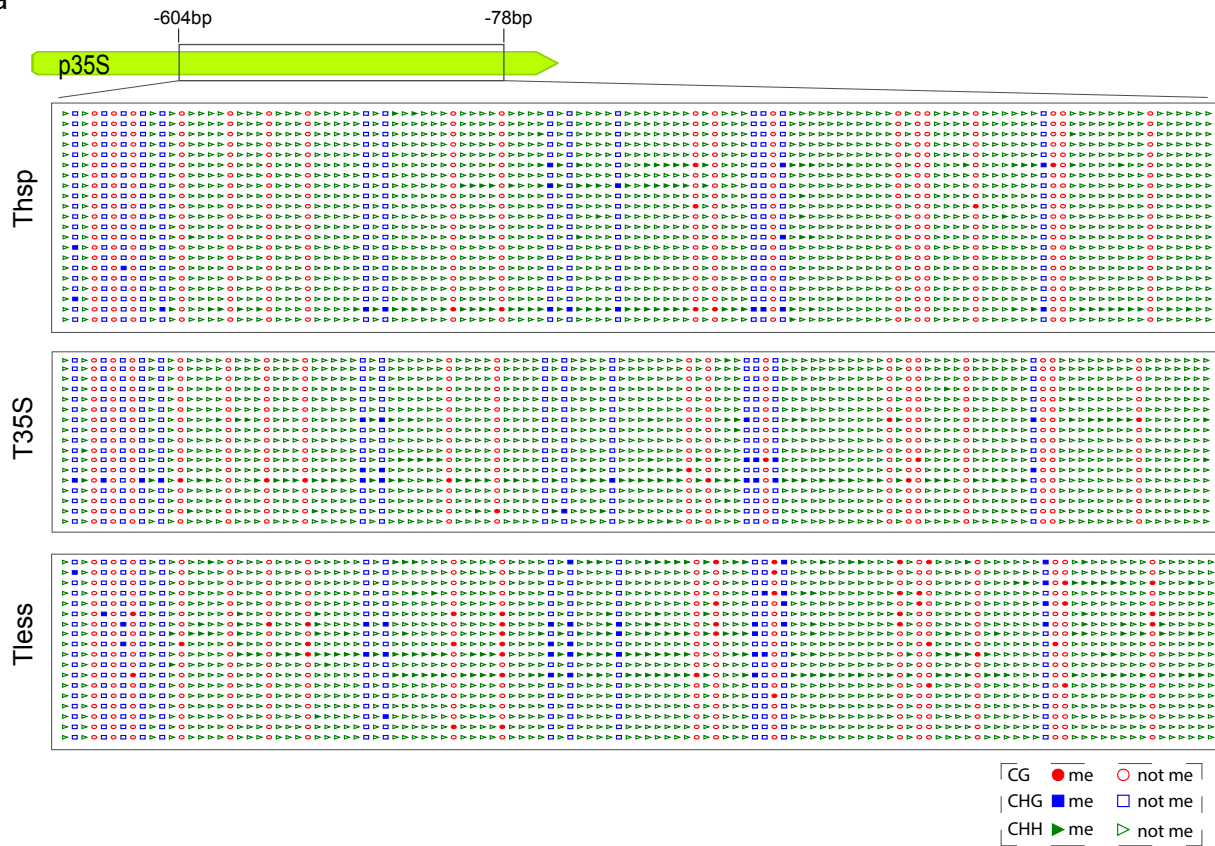

b

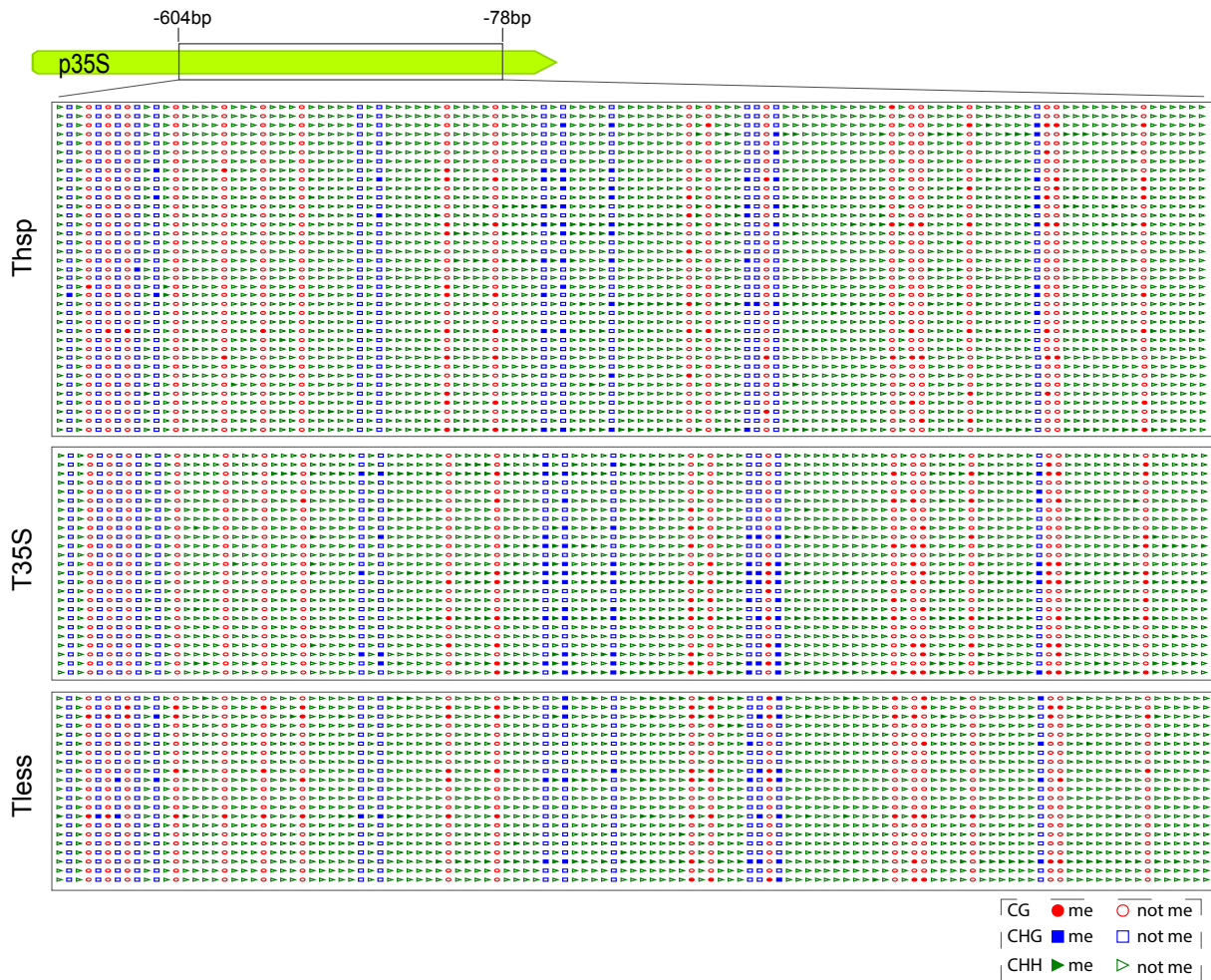

C

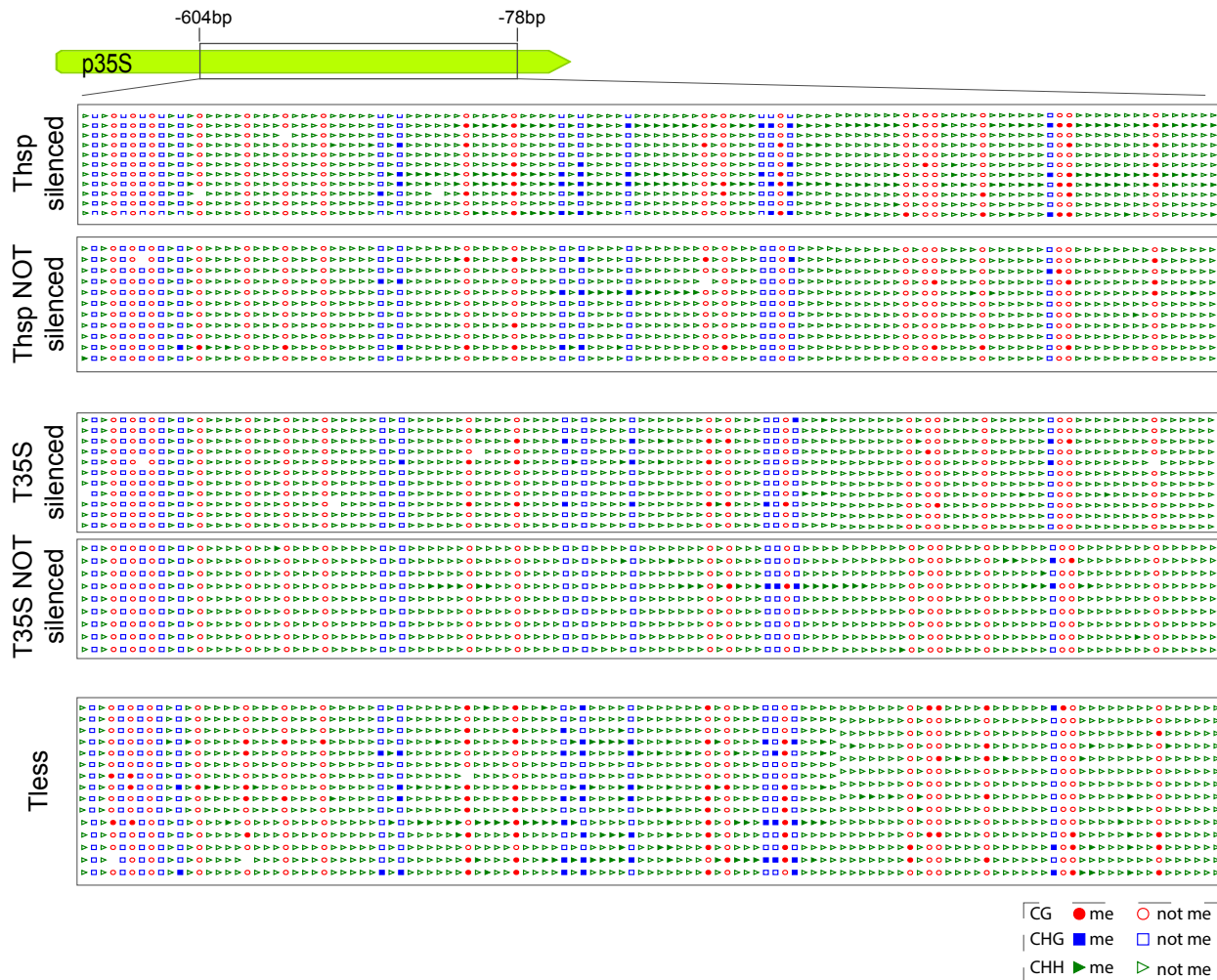

Supplement: Supplementary file 2 — Additional file 2: Figure S2. DNA methylation of the 35S CaMV promoter. a) Graphical output of the promoter methylation analysis (CyMate software) in 10 day-old seedlings. b) Graphical output of the promoter methylation analysis (CyMate software) in a pool of mature leaves from each construction. c) Graphical output of the promoter methylation analysis (CyMate software) in pools of mature leaves from silenced and not silenced lines from each construction. Red circles represent CG sites, blue squares represent CHG sites and green triangles represent CHH sites. Filled symbols indicate methylated cytosines while empty ones represent non methylated cytosines. [file 13104_2018_3649_MOESM2_ESM.pdf]
