# Supplementary material for: Effect of transcription terminator usage on the establishment of transgene transcriptional gene silencing
Source: BMC Res Notes. 2018 Jul 28;11:511. doi: 10.1186/s13104-018-3649-2 (PMC6064074; doi:10.1186/s13104-018-3649-2)

A

Thsp line	Segregation (resistant:sensitive)	Segregation ratio
**1**	54:6	9
**2**	53:6	8,83
**3**	48:11	4,36
**4**	43:18	2,38
**8**	43:14	3,07
**9**	40:18	2,22
**11**	57:3	19
**15**	47:9	5,22
**17**	44:13	3,38
**19**	44:13	3,38
**20**	46:14	3,28
**21**	56:4	14
**22**	44:16	2,75

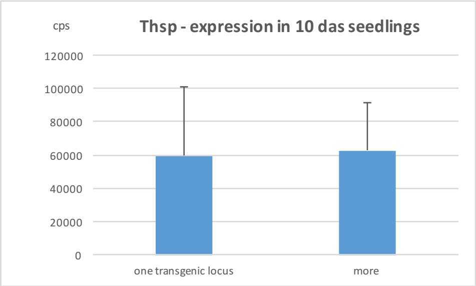

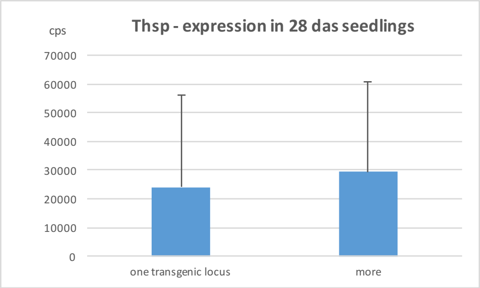

B

T35S line	Segregation (resistant:sensitive)	Segregation ratio
**1**	49:11	4,45
**2**	46:7	6,57
**3**	47:10	4,7
**4**	45:14	3,21
**5**	32:25	1,28
**7**	44:5	8,8
**10**	45:15	3
**12**	47:11	4,27
**17**	46:12	3,83
**19**	43:13	3,3
**20**	43:16	2,68
**21**	48:10	4,8
**22**	44:16	2,75

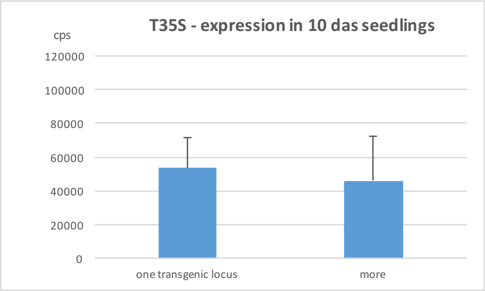

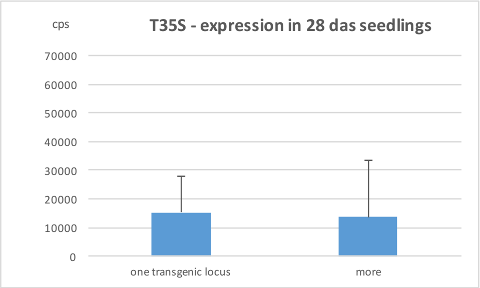

C

Tless lines	Segregation (resistant:sensitive)	Segregation ratio
**1**	51:7	7,28
**2**	44:12	3,66
**3**	47:14	3,35
**5**	36:20	1,8
**10**	45:13	3,46
**12**	46:13	3,53
**16**	51:7	3,53
**17**	35:22	1,59
**19**	26:32	0,81
**23**	45:14	3,21
**24**	46:13	3,53
**25**	46:11	4,18
**28**	40:18	2,22

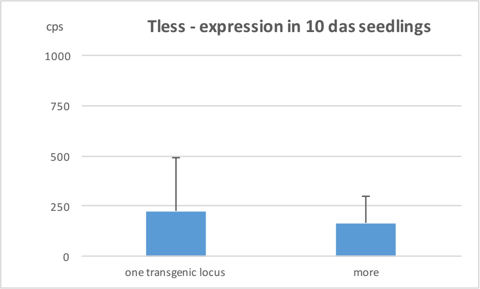

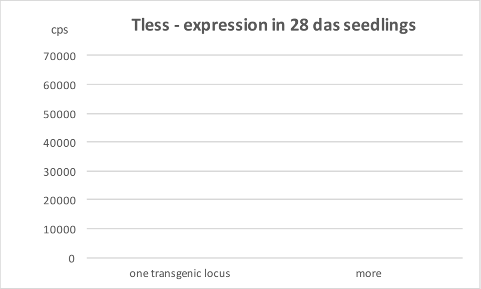

Supplement: Supplementary file 3 — Additional file 3: Figure S3. T2 HygroB resistance segregation ratios and relationship to LUC expression. A) Data for Thsp lines. B) Data for T35S lines. C) Data for Tless lines. Note that lines with a segregation ratio < 4 (red) were considered to have only one transgenic locus. [file 13104_2018_3649_MOESM3_ESM.pdf]
